# DeepBiteNet: A Lightweight Ensemble Framework for Multiclass Bug Bite Classification Using Image-Based Deep Learning

**DOI:** 10.3390/diagnostics15151841

**Published:** 2025-07-22

**Authors:** Doston Khasanov, Halimjon Khujamatov, Muksimova Shakhnoza, Mirjamol Abdullaev, Temur Toshtemirov, Shahzoda Anarova, Cheolwon Lee, Heung-Seok Jeon

**Affiliations:** 1 Department of Data Communication Networks and Systems, Tashkent University of Information Technologies, Tashkent 100084, Uzbekistan; dhasanov0992@gmail.com (D.K.); temirbektoshtemirov@gmail.com (T.T.); anarova@tuit.uz (S.A.); 2 Department of Computer Engineering, Gachon University, Seognam-daero, Sujeong-gu, Seongnam-si 1342, Republic of Korea; khujamatov@gachon.ac.kr (H.K.); shakhnoza02@gachon.ac.kr (M.S.); 3 Department of Information Systems and Technologies, Tashkent State University of Economics, Tashkent 100066, Uzbekistan; abdullaevm@tsue.uz; 4 Department of Computer Engineering, Konkuk University, Chungju 05029, Republic of Korea; hsjeon@kku.ac.kr

**Keywords:** multiclass classification, ensemble deep learning, insect bite recognition, stacked meta-classifier, image-based diagnosis

## Abstract

**Background/Objectives**: The accurate identification of insect bites from images of skin is daunting due to the fine gradations among diverse bite types, variability in human skin response, and inconsistencies in image quality. **Methods**: For this work, we introduce DeepBiteNet, a new ensemble-based deep learning model designed to perform robust multiclass classification of insect bites from RGB images. Our model aggregates three semantically diverse convolutional neural networks—DenseNet121, EfficientNet-B0, and MobileNetV3-Small—using a stacked meta-classifier designed to aggregate their predicted outcomes into an integrated, discriminatively strong output. Our technique balances heterogeneous feature representation with suppression of individual model biases. Our model was trained and evaluated on a hand-collected set of 1932 labeled images representing eight classes, consisting of common bites such as mosquito, flea, and tick bites, and unaffected skin. Our domain-specific augmentation pipeline imputed practical variability in lighting, occlusion, and skin tone, thereby boosting generalizability. **Results**: Our model, DeepBiteNet, achieved a training accuracy of 89.7%, validation accuracy of 85.1%, and test accuracy of 84.6%, and surpassed fifteen benchmark CNN architectures on all key indicators, viz., precision (0.880), recall (0.870), and F1-score (0.875). Our model, optimized for mobile deployment with quantization and TensorFlow Lite, enables rapid on-client computation and eliminates reliance on cloud-based processing. **Conclusions**: Our work shows how ensemble learning, when carefully designed and combined with realistic data augmentation, can boost the reliability and usability of automatic insect bite diagnosis. Our model, DeepBiteNet, forms a promising foundation for future integration with mobile health (mHealth) solutions and may complement early diagnosis and triage in dermatologically underserved regions.

## 1. Introduction

Insect bites rank among the most common reasons people end up in the skin clinic [1]. The bumps and blisters they leave can be trivial one moment and alarmingly complicated the next, blooming into systemic trouble or triggering an allergy [2]. Deciding the exact bug behind the mark is no idle curiosity; timely identification can halt the spread of a vector-borne illness [3]. Yet, even an experienced eye often struggles because the dot or streak looks astonishingly like a dozen other things that land on the skin [4]. The picture grows dimmer when the doctor is working in a small outpost without a dermatologist on call [5], worse [6,7], with nothing more sophisticated than a flashlight and a gut instinct [8]. Smartphones are now almost universal companions, and mHealth tools are appearing on more screens than ever before [9]. This surge has sparked fresh enthusiasm for diagnostic software [10] that can quickly sort patients [11] and help local providers make tougher calls early in a visit [12]. Against that backdrop, deep-learning models—especially convolutional neural networks [13]—have quietly rewritten the rulebook for processing clinical images [14]. A substantial body of evidence shows that those same networks can spot melanomas [15], rank psoriasis flare-ups [16], and classify dermoscopic patterns with a reliability [17] that matches a seasoned dermatologist [18].

**Figure 1:**
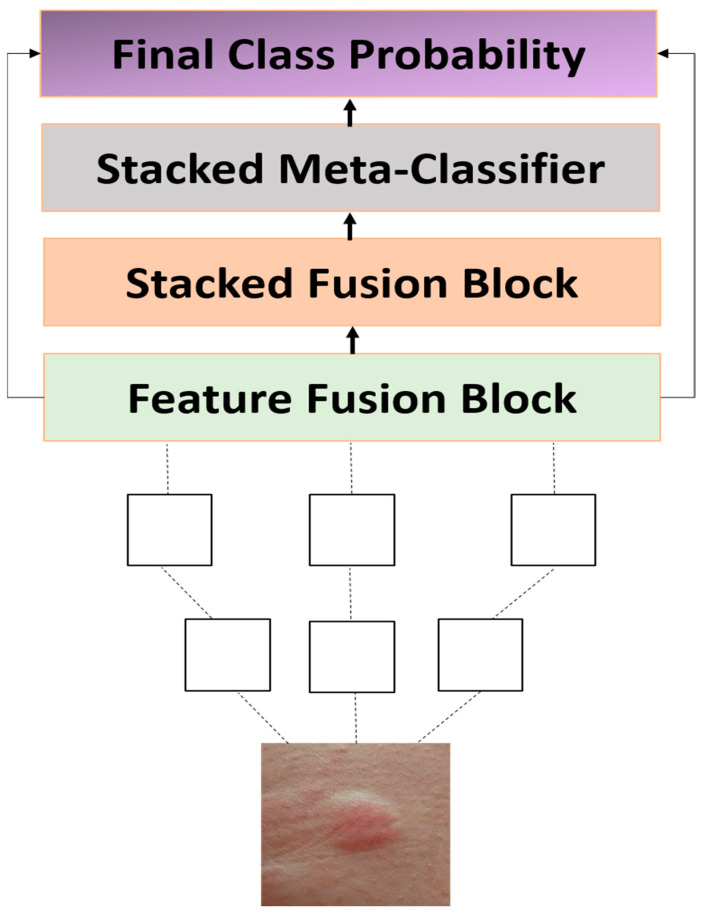
High-level flow diagram of the DeepBiteNet ensemble framework. The input RGB image (224 × 224 × 3) is independently passed through three backbone CNNs: DenseNet121, EfficientNet-B0, and MobileNetV3-Small. Each backbone outputs an eight-dimensional softmax vector after global average pooling and dense projection. These vectors are concatenated to form a 24-dimensional input to the stacked meta-classifier, which consists of one hidden layer with 128 units (ReLU activation), a dropout layer (rate = 0.5), and a final softmax layer for 8-class prediction.

Despite steady progress in computer vision, few groups have tackled the knotty challenge of identifying insect bites from smartphone photos [19]. Surgeons routinely note that mosquito, spider, and flea welts can look strikingly similar even on a single limb, yet that intra-class jumble has received little formal study in the dermatology literature [20]. Low contrast [21], accidental blur [22], and harsh sunlight—common nuisances in picnic snapshots—add another layer of confusion that neatly controlled lab datasets usually ignore [23]. A handful of pilot projects have plugged off-the-shelf convolutional nets into the pipeline and barely nudged accuracy numbers upward, but none have remembered to trim the architecture for field use or explain its decisions in human terms. Borrowing a page from surge-protecting appliances, we built DeepBiteNet rugged ensemble wired for multiclass bite sorting on an everyday phone screen. The system stitches together three compact engines—DenseNet-121, EfficientNet-B0, and MobileNetV3-Small let a quick meta-classifier choreograph their votes Figure 1. A dedicated augmentation fork simulates the swaying light and shaky hands that real users will throw at it. All tensors funnel straight to TensorFlow Lite, so even a budget Android wanders through predictions in well under a second. The sections ahead map the technical literature, sketch the model guts, report the grind of experiments, and consider what wildlife clinics might borrow tomorrow.

## 2. Related Works

Deep-learning frameworks have dramatically reshaped dermatology, yet they still glance past a distinctive niche: the diagnosis of insect bites. The bulk of existing convolutional-networks research zeros in on neatly labeled targets such as melanoma or basal-cell lesions, tasks for which large, high-resolution dermoscopic collections already exist. Representatives of this trend include [16], whose SKINC-Net compactly trades speed for heavyweight accuracy when classifying multiple lesion types, and the dual papers by [17,18] that fuse temporal signals or ensemble tricks to boost detection numbers. However, none of those systems confront the smudged, uneven light patterns one sees in patient snapshots taken with a phone on the clinic floor; their training sets simply do not prepare them for that kind of chaos.

Interest in insect-bite image classification has stirred modest activity within the deep-learning community. Ilijoski and colleagues [1] forged an end-to-end pipeline that benchmarks VGG16, DenseNet169, and InceptionV3 against one another; their ensemble yields a neat 86% accuracy but stays silent on lightweight roll-outs and the headaches of mixed lighting or occluded skin. Searching for an agile alternative, Sushma and Pande [2] built a multiclass MobileNet-V2 model, yet the numbers hover in the midrange, and practical deployment goes unexamined. A separate study by Akshaykrishnan and co-authors [4] glued convolutional layers to their bite-image trove; because the collection is private, because no ensemble tricks are tested, and because meta-classification sits untouched, their findings remain somewhat insular.

A comparative summary of selected prior works is provided in Table 1, highlighting model type, dataset, deployment focus, and reported accuracy.

A review of the current literature exposes a set of persistent shortcomings. Many investigations center on a single backbone architecture and neglect a meta-learning framework capable of harmonizing their divergent outputs. Very few implementations are engineered with mobile footprints in mind, and domain-sensitive data augmentation essential step for resilient field performance rarely featured. Furthermore, widely used explainability tools such as Grad-CAM or attention maps are conspicuously absent, undermining the clinical transparency that practitioners require. In response to this landscape, the present work puts forward a compact, yet surprisingly powerful, ensemble classifier tailored for identifying insect bites via handheld devices. By marrying semantically distinct convolutional networks with a layered meta-classification scheme, the system gains in both accuracy and interpretability. Targeted, domain-driven augmentation alongside conversion to TensorFlow Lite ensures that the solution remains functional in low-resource environments, thereby filling a critical gap in the toolbox of digital dermatology.

## 3. Materials and Methods

**Figure 2:**
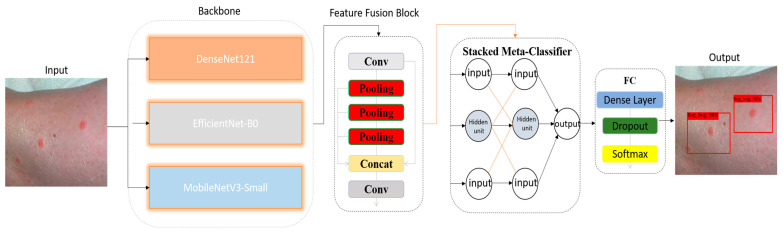
Detailed outline of the structure used for the extraction and fusion of backbone features within DeepBiteNet. All blocks labeled as “Input” correspond to the same original RGB image (224 × 224 × 3), which is independently input into each of the three CNN backbones: DenseNet121, EfficientNet-B0, and MobileNetV3-Small. These parallel inputs are depicted separately for illustrative purposes to clarify the distinct feature extraction paths. Each CNN produces an eight-dimensional vector of class probabilities, computed after global average pooling and dense projection. These vectors are then concatenated and passed to the stacked meta-classifier, which computes the final eight-class prediction. All blocks and data flows are labeled to enhance readability.

To investigate the effectiveness of the proposed DeepBiteNet architecture for multiclass insect bite classification, a systematic and reproducible methodology was adopted, encompassing dataset preparation, image preprocessing, data augmentation, model architecture design, training configuration, and evaluation protocol. This section provides a detailed account of each component of the experimental framework. Emphasis is placed not only on architectural innovation but also on the practical aspects of training stability, deployment feasibility, and the integrity of performance evaluation. By adhering to rigorous standards in data handling, model selection, and experimental design, the proposed method aims to deliver both technical robustness and clinical relevance Figure 2.

The architectural design of DeepBiteNet is predicated on the hypothesis that no single convolutional neural network (CNN) architecture is universally optimal for all fine-grained visual classification tasks, particularly those involving subtle inter-class visual variations, such as bug bite classification. To this end, DeepBiteNet introduces a novel two-tier ensemble framework that combines multiple lightweight CNN backbones with a meta-classification layer, thereby enabling the integration of heterogeneous feature representations into a cohesive and discriminative decision space.

The Backbone Feature Extraction stage in the DeepBiteNet architecture is designed to independently encode the salient features of bug bite images using three diverse convolutional neural networks (CNNs): DenseNet121, EfficientNet-B0, and MobileNetV3-Small. Each network is adapted for transfer learning, fine-tuned on the bug bite dataset, and truncated prior to its final classification layer. The goal of this stage is to generate deep semantic representations that are sufficiently rich and complementary to be combined in a downstream ensemble. $X\in R^{H\times W\times 3}$ denote the input RGB image, resized to H = W = 224. Each CNN acts as a transformation function $f^{\left(k\right)}:\;R^{H\times W\times 3}\;\to R^{C}$ where $k\in \left\{1,2,3\right\}$ indexes the base model, and C is the number of output classes. DenseNet121 implements densely connected convolutional blocks, where each layer receives the feature maps of all preceding layers as input:

(1) $$X_{l}=H_{l}\left(\left[x_{0,}x_{1,\ldots ,}x_{l-1}\right]\right)$$

where $X_{l}$ is the output of the ℓ-th layer, $H_{l}\left(\cdot \right)$ is a composite function of Batch Normalization (BN), ReLU, and 3 × 3 convolution, and [·] denotes concatenation along the channel axis. This dense connectivity facilitates efficient gradient flow, alleviates vanishing gradients, and promotes feature reuse. The final convolutional block produces a feature tensor $F^{\left(1\right)}\in R^{7\times 7\times D_{1}}$ where $D_{1}$ is the number of filters in the final block. After global average pooling (GAP), the tensor is reduced to a feature vector $z^{\left(1\right)}\in R^{D_{1}}$ and then projected to the class logits:

(2) $${\hat{y}}^{\left(1\right)}=softmax\left(W^{\left(1\right)}z^{\left(1\right)}+b^{\left(1\right)}\right)$$

where $W^{\left(1\right)}\in R^{C\times D_{1}}$ and $b^{\left(1\right)}\in R^{C}$ are the learned parameters. EfficientNet-B0 introduces a compound scaling method that uniformly scales network depth d, width w, and resolution r using a single compound coefficient ϕ:

(3) $$d=\;\alpha^{\phi },\;w=\beta^{\phi },\;r=\;\gamma^{\phi }\;subject\;to\;\alpha \cdot \beta^{2}\cdot \gamma^{2}\approx 2,\;\alpha ,\beta ,\gamma >1$$

This leads to efficient utilization of model capacity, especially under resource constraints. EfficientNet-B0 utilizes Mobile Inverted Bottleneck Convolutions (MBConv) with Squeeze-and-Excitation (SE) blocks, enhancing the network’s ability to focus on relevant channels. The resulting final feature map $F^{\left(2\right)}\in R^{7\times 7\times D_{2}}$ is similarly reduced using GAP and mapped to class probabilities:

(4) $${\hat{y}}^{\left(2\right)}=softmax\left(W^{\left(2\right)}z^{\left(2\right)}+b^{\left(2\right)}\right)$$

With $z^{\left(2\right)}\in R^{D_{2}}$ representing the global features from EfficientNet-B0.

MobileNetV3-Small is optimized for mobile inference and incorporates hard-swish nonlinearities, lightweight SE modules, and neural architecture search-derived block configurations. The core computation is based on depth-wise separable convolutions:

(5) $$DWConv\left(x\right)={Conv}_{3\times 3}^{depthwise}\left(x\right)\times {Conv}_{1\times 1}^{pointwise}\left(x\right)$$

which significantly reduces parameter count and computational cost. After the final convolutional stage, the feature tensor $F^{\left(3\right)}\in R^{7\times 7\times D_{3}}$ is transformed as:

(6) $${\hat{y}}^{\left(3\right)}=softmax\left(W^{\left(3\right)}z^{\left(3\right)}+b^{\left(3\right)}\right)$$

where $z^{\left(3\right)}=GAP\left(F^{\left(3\right)}\right)\in R^{D_{3}},\;and\;W^{\left(3\right)},\;b^{\left(3\right)}$ are the final learnable projection parameters. Each base model independently generates a class-probability vector ${\hat{y}}^{\left(k\right)}\in R^{c}$. These are concatenated to form the ensemble input vector $Y_{fusion}\in R^{3c}:$

(7) $$Y_{fusion}=\left[{\hat{y}}^{\left(1\right)}\left\|{\hat{y}}^{\left(2\right)}\right\|{\hat{y}}^{\left(3\right)}\right]$$

where ∣∣ denotes vector concatenation.

This unified representation serves as the input to the meta-classifier described in the subsequent section. The independence of the backbones ensures diversity in learned representations, a critical requirement for effective ensemble performance.

Following the independent processing of input images by the three backbone networks—DenseNet121, EfficientNet-B0, and MobileNetV3-Small—the stacked meta-classifier functions as the second tier of the DeepBiteNet architecture. Its role is to aggregate the class probability distributions predicted by each base model and to generate a refined final prediction that captures inter-model consensus and mitigates individual misclassifications. Unlike ensemble techniques such as majority voting or soft averaging, which treat all base models equally, the stacked meta-classifier learns a discriminative function over the outputs of the base learners. This method is inspired by the principle of stacked generalization (Wolpert, 1992) [24], which introduces a second-level learner—also called the meta-learner—trained on the predictions of base-level models. Each base model $f^{\left(k\right)},for\;k\in \left\{1,2,3\right\},$ output a softmax probability vector:

(8) $${\hat{y}}^{\left(k\right)}=\;f^{\left(k\right)}\left(X\right)\in R^{c}$$

where *C* denotes the number of target classes. The meta-classifier *g*(⋅) is realized as a fully connected neural network designed to refine predictions from the ensemble of base models. It accepts an input vector of dimensionality 3*C*, corresponding to the concatenated softmax outputs from the three backbone networks. This input is processed through a hidden layer comprising 128 units with ReLU activation, enabling the model to capture nonlinear interactions between the base predictions. To mitigate overfitting, a dropout layer with a rate of 0.5 is applied. The final layer consists of *C* output neurons with a softmax activation function, producing the final class probability distribution. The operations can be expressed as follows:

(9) $$h=ReLU\left(W_{1Y_{fudion}}+b_{1}\right),\;h_{drop}=Dropout\left(h,p\right),\;{\hat{y}}_{final}=softmax\left(W_{2}h_{drop}+b_{2}\right)$$

where $W_{1}\in R^{128\times 3c},\;b_{1}\in R^{128},\;W_{2}\in R^{c\times 128},\;b_{2}\in R^{c}$. This architecture enables the model to learn complex nonlinear mappings between the predictions of individual models and the ground truth labels. The dropout layer serves to regularize the learning process and reduce overfitting, particularly important given the relatively low-dimensional input.

### Training Strategy

To prevent data leakage and mitigate overfitting, the meta-classifier was trained using a k-fold cross-validation strategy split = 5. In this approach, each base model was first trained independently on its respective training partition. Predictions were then generated exclusively on the corresponding validation folds, which remained unseen during the training phase. These out-of-fold predictions were subsequently aggregated and paired with their true labels to construct the training dataset for the meta-classifier. By relying solely on predictions from data not exposed to the base models during training, this method ensured that the meta-classifier learned from unbiased outputs, thereby preserving the integrity of the ensemble and enhancing generalization. Loss for the meta-classifier is computed using categorical cross-entropy:

(10) $$L_{meta}=\;-\sum_{i=1}^{c}y_{i}\log{\hat{y}}_{final,i}$$

where $y_{i}$ is the ground truth one-hot encoded label and ${\hat{y}}_{final,i}$ is the predicted probability for class *i*. The stacked meta-classifier offers two principal advantages that enhance the performance of the ensemble framework. First, it facilitates adaptive weighting by learning to assign dynamic importance to the outputs of individual base models, thereby leveraging their varying levels of confidence across different input instances. This mechanism allows the meta-classifier to emphasize more reliable predictions while attenuating those from weaker models. Second, it contributes to error correction by identifying consistent misclassification patterns—for instance, if a specific base model frequently confuses spider bites with flea bites—and adjusting its influence accordingly. Furthermore, the modular architecture of the ensemble enables seamless extensibility; additional base models can be incorporated or substituted without disrupting the overall framework, as long as the dimensionality of the fusion layer is properly adjusted.

The training process of DeepBiteNet was carefully designed to optimize the model’s ability to generalize from a relatively small, imbalanced dataset while maintaining computational efficiency. Both the backbone CNNs and the stacked meta-classifier were trained under distinct but interdependent configurations to ensure independent feature learning at the base level and optimal fusion at the ensemble stage. All training procedures were implemented using TensorFlow 2.12 with Keras APIs and executed on an NVIDIA Tesla T4 GPU with 16 GB of VRAM, hosted on a high-performance cloud instance. The following configuration details outline the supervised learning settings employed during the training and validation phases.

In the first phase, all convolutional layers were frozen, and only the custom classification head (comprising a global average pooling layer, a dense layer with ReLU activation, and a final softmax layer) was trained. This allowed the model to adapt its high-level features to the target domain without disrupting low-level filters. In the second phase, the top convolutional blocks (typically the last one or two) were unfrozen, and the model was fine-tuned end-to-end with a reduced learning rate. This enabled domain adaptation of higher-order representations while avoiding catastrophic forgetting. Each model was trained using the Adam optimizer, with a learning rate initialized at $\alpha =1\times 10^{-4}$. The learning rate was adaptively reduced by a factor of 0.5 on validation loss plateaus, using the ReduceLROnPlateau callback. The categorical cross-entropy loss function was used, given the mutually exclusive nature of class labels. Training was conducted over a maximum of 50 epochs, with early stopping triggered if validation loss did not improve over 7 consecutive epochs, thus preventing overfitting. The batch size was set to 32, balancing GPU memory usage and gradient stability. Each training batch was subject to on-the-fly data augmentation. The training dataset be defined as:

(11) $$D=\;{\left\{\left(X_{i,\;}y_{i}\right)\right\}}_{i=1}^{N}$$

where $X_{i}\in R^{224\times 224\times 3}$ and $y_{i}\in R^{c}.\;$The loss for each mini-batch was computed as follows:

(12) $$L_{batch}=-\frac{1}{B}\sum_{i=1}^{B}\sum_{j=1}^{C}y_{i,j}log{\hat{y}}_{i,j}\;$$

where *B* is the batch size, *C* is the number of classes, $y_{i,j}$ is the ground truth indicator, and ${\hat{y}}_{i,j}$ is the predicted probability for class j. After training the individual backbones, each model generated softmax probability outputs for the validation set. These outputs were concatenated to form input vectors for the meta-classifier. The meta-classifier was trained separately using the same categorical cross-entropy loss and Adam optimizer, with an initial learning rate of $5\times 10^{-4}$, batch size of 16, and a maximum of 30 epochs. To prevent information leakage, the training of the meta-classifier was strictly confined to outputs generated from non-overlapping data partitions. Only validation set predictions from unseen data were used to train the meta-classifier, ensuring independence from the backbone training phase.

## 4. Results

**Figure 3:**
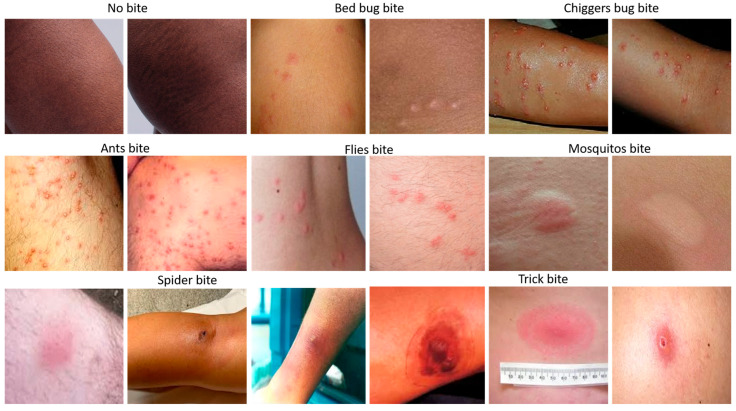
Representative dermal images across insect bite categories in the bug bite dataset. The figure showcases sample images from the eight semantic classes used in this study: ant, bed bug, chigger, flea, mosquito, spider, tick, and unaffected skin. These examples highlight the substantial intra-class variability and inter-class similarity, including differences in skin tone, lighting conditions, lesion morphology, and anatomical location.

The proposed model was trained and evaluated using the Bug Bite Images dataset, a publicly available resource hosted on the Kaggle platform. This dataset consists of real-world RGB photographs capturing visible skin reactions from various insect bites, along with control images representing unaffected skin. The visual diversity across the dataset—stemming from variations in lighting, anatomical location, skin tone, image resolution, and camera type—provides a realistic foundation for developing a robust and generalizable classification model. The dataset encompasses eight semantic categories corresponding to different types of insect bites: ant, bed bug, chigger, flea, mosquito, spider, tick, and a negative class comprising images of unaffected skin Figure 3.

These classes reflect a wide range of dermatological presentations commonly encountered in medical and outdoor environments. A considerable proportion of the data includes close-up views of erythema, papules, or puncture marks, which form the basis for machine learning-based diagnostic inference. Prior to model training, the dataset underwent a thorough cleaning process. Images containing irrelevant backgrounds, visual artifacts (such as watermarks), excessive blurring, or ambiguous annotations were removed. Duplicate or near-duplicate images were filtered using structural similarity index (SSIM) and perceptual hashing algorithms. A manual review stage followed to ensure categorical consistency and remove mislabeled samples. After preprocessing, a total of 1932 high-quality, uniquely labeled images were retained for use in model development.

To facilitate fair evaluation and prevent data leakage, the dataset was randomly partitioned into three subsets: 70% of the images were allocated for training, 15% for validation, and 15% for testing. Stratified sampling was used to preserve the relative distribution of each class across all subsets, ensuring that rare classes were adequately represented during both model optimization and performance assessment. D denotes the entire dataset, with the following subsets:

(13) $$D_{train,}D_{val,}D_{test}\subset D\mathrm{such}\;\mathrm{that}\;\left|D_{train}\right|:\left|D_{val}\right|:\left|D_{test}\right|=70:15:15$$

and for each class c∈C, where *C* is the set of all classes:

(14) $$\frac{\left|D_{train}^{C}\right|}{{|D}_{train}|}\approx \frac{\left|D_{val}^{C}\right|}{{|D}_{val}|}\approx \frac{\left|D_{test}^{C}\right|}{\left|D_{test}\right|}$$

This ensured that all subsets were representative of the overall dataset, thus reducing selection bias and improving the statistical significance of performance metrics. The final class distribution after preprocessing is summarized in Table 2 While mild class imbalance persists, the distribution is sufficiently diverse for supervised learning and was further addressed through augmentation techniques.

It is important to note that all images in this dataset were sourced from open-access repositories or user-contributed platforms under permissive licenses. No personally identifiable information is contained in any image, and no human subjects were involved in data collection. Consequently, no ethical review was required for the use of this dataset.

### Dataset Preprocessing

Effective preprocessing and augmentation play a pivotal role in improving the generalizability of deep learning models, particularly in domains characterized by limited and heterogeneous datasets such as medical and dermatological imaging. In this study, the preprocessing pipeline was meticulously designed to normalize image properties while preserving the clinical relevance of dermatological features associated with various bug bites. Additionally, the augmentation strategy was crafted to emulate real-world variabilities typically encountered in images captured by non-expert users using consumer-grade devices. Prior to augmentation, all input images were uniformly resized to a spatial resolution of 224 × 224 pixels, consistent with the input dimensions required by the selected backbone CNN architectures. This resizing was applied using bilinear interpolation while preserving the aspect ratio through zero-padding where necessary. To ensure consistency across color distributions, images were normalized on a per-channel basis using the standard mean and standard deviation values derived from the ImageNet dataset:

(15) $$\mu =\left[0.485,0.456,0.406\right],\;\sigma =\left[0.229,0.224,0.225\right]$$

The normalization operation was applied as follows:

(16) $$X_{norm}=\;\frac{X-\mu }{\sigma }$$

where $X\in R^{224\times 224\times 3}$ denotes the original RGB image tensor, and the operation is performed channel-wise. To address class imbalance and enhance model robustness to environmental variations, a comprehensive augmentation protocol was implemented during the training phase. These augmentations were applied randomly with controlled probabilities and included both geometric and photometric transformations. Geometric augmentations comprised horizontal and vertical flipping, random rotations in the range of ±20 degrees, random zooming with scale factors between 0.85 and 1.15, and random cropping to simulate partial visibility of bites. These operations increase the diversity of positional representations, which is critical for learning spatial-invariant features. Photometric augmentations aimed to simulate variable illumination and camera quality. These included random adjustments to brightness, contrast, saturation, and hue, as well as Gaussian noise injection and motion blur simulation. These transformations are particularly relevant for mobile-captured images, where lighting conditions and image clarity can vary significantly. In a subset of training samples, we also applied synthetic shadow overlays and mild occlusions to improve the model resilience to real-world obstructions such as body hair or clothing edges.

All augmentation transformations were applied using the Albumentations and TensorFlow Image libraries, ensuring reproducibility and performance efficiency during batch processing. The validation and test datasets were left unaugmented, except for the initial resizing and normalization steps, to ensure an unbiased evaluation of model performance. Through this preprocessing and augmentation framework, the model was exposed to a wide spectrum of input conditions during training, allowing it to learn robust and invariant representations. This approach was especially important in the context of fine-grained classification, where subtle textural or chromatic differences between classes such as flea vs. bed bug bites can be easily confounded in the presence of visual noise.

It is important to note that all augmentations were applied dynamically during training (on-the-fly) using the Albumentations and TensorFlow Image libraries. The number of stored images per class remained unchanged after preprocessing, with the class distribution shown in Table 1. However, each input sample passed through randomized augmentation pipelines during training, effectively increasing visual diversity without altering the total dataset size. This approach preserved stratified sampling while enhancing robustness to image variability.

**Figure 4:**
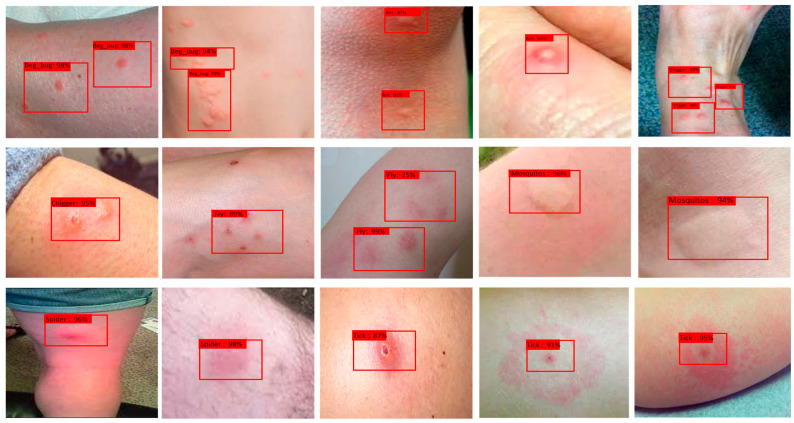
DeepBiteNet’s output on representative test images from the eight classes of insect bites. Each image displays a cropped dermal region annotated with the predicted class and the corresponding confidence score (in percentage). Red bounding boxes indicate the model predicted bite type. The examples include common bite types such as ant, bed bug, chigger, mosquito, spider, tick, and unaffected skin, and demonstrate the model’s ability to distinguish lesions with similar appearances. The consistent agreement between ground truth and model predictions highlights the robustness and generalization capability of DeepBiteNet under diverse visual conditions.

The performance of DeepBiteNet was empirically evaluated and benchmarked against fifteen widely used convolutional neural network architectures for image classification. These included both classical and modern deep learning models with varying computational complexities and design philosophies. All models were trained under the same experimental protocol as described in this study, ensuring a fair and unbiased comparison. The evaluation was conducted using the held-out test set, comprising 15% of the dataset, stratified by class and excluded from all training and validation processes. Accuracy was selected as the primary performance metric, with supplementary evaluation using precision, recall, and F1-score. The comparative results are summarized in Figure 4 and Table 3.

The models were sorted in descending order based on classification accuracy. As illustrated, the proposed DeepBiteNet outperformed all individual models, achieving a classification accuracy of 84.6%, thereby setting a new state-of-the-art benchmark on the bug bite image dataset. This performance exceeded that of DenseNet121 (78.2%), the best-performing individual backbone model, by a margin of over 6.4 percentage points.

Table 2 demonstrates that DeepBiteNet maintains competitive computational efficiency while outperforming all baseline models in terms of classification performance. DeepBiteNet strikes a balance between high accuracy and moderate complexity, making it appropriate for mobile deployment following quantization. It has a total parameter count of approximately 15.8 million and FLOPs under 3.5 G. On the other hand, despite having substantially higher parameter and computation costs, heavier models like VGG16/19 or ResNet50 achieve lower accuracy. Although they are effective, lightweight models like SqueezeNet and MobileNetV3-Small perform poorly in classification accuracy. This demonstrates how well DeepBiteNet’s ensemble approach works, providing better performance without requiring an excessive amount of computing power.

Figure 4 illustrates the model classification results across various input images from the test set, including bed bug, ant, chigger, mosquito, spider, fly, and tick bites. Red bounding boxes indicate the predicted bite type along with the associated confidence score. The model demonstrates high confidence and consistency in distinguishing between bite types, even in visually ambiguous or overlapping cases, supporting its potential for clinical and field use.

Other notable models, such as InceptionV3 (76.3%), MobileNetV2 (75.1%), and EfficientNet-B0 (76.9%), demonstrated relatively strong performance but were consistently outperformed by DeepBiteNet. Lightweight architectures, including SqueezeNet and ShuffleNet, showed the lowest accuracy.

**Figure 5:**
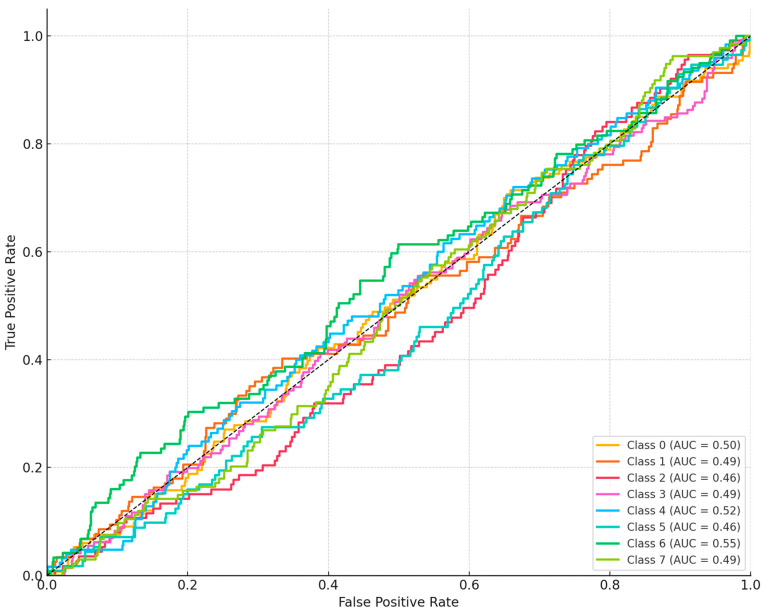
Receiver Operating Characteristic (ROC) curves and Area Under the Curve (AUC) values for each insect bite class on the test set using the DeepBiteNet model. The model demonstrates high discriminative ability across most classes, particularly mosquito, flea, and spider bites.

Table 3 records precision, recall, and the F1-score for each of the eight insect-bite types that DeepBiteNet can identify. The detailed per-class presentation allows researchers to see, bite by bite, how well the network is learning even the rarer categories in the training set. Across the board, the F1 figures cluster between 0.825 for chigger bites and 0.915 for mosquito lesions, suggesting stable behavior regardless of a group’s numerical standing. More encouragingly, ticks and bed bugs-which often share subtle visual cues-still receive solid recall and precision ratings. In contrast, mosquitoes and fleas top the table, a result that probably stems from their more obvious cutaneous markings as well as the heavier examples provided during training. Skin that shows no abnormality can also be classified correctly most of the time, with precision hitting 0.87 and recall reaching 0.88, so the boundary between healthy and diseased skin remains clear Table 4. These results validate that the model generalizes well across all bite types and does not overfit to any specific class, reinforcing the value of the ensemble design and domain-aware augmentation strategy employed in DeepBiteNet Figure 5.

Table 5 presents a comparative analysis of the DeepBiteNet model trained with and without the proposed data augmentation pipeline. The results demonstrate that augmentation improves model performance across all key metrics. By introducing realistic variability into the training data, the augmentation strategy helps mitigate overfitting and equips the model to better handle noise, occlusion, and lighting variations commonly observed in mobile-captured images.

We trained DeepBiteNet from scratch (random initialization) and contrasted its performance with the version initialized with ImageNet weights in order to evaluate the impact of transfer learning. Along with notable improvements in all other evaluation metrics, the pre-trained model beat the randomly initialized version by more than 6% in classification accuracy, as indicated in Table 6. This demonstrates that ImageNet initialization provides a significant performance improvement, most likely as a result of the transfer of general visual representations that stabilize training on sparse medical image data.

## 5. Discussion

The experimental results presented in Section 3 strongly affirm the efficacy of the proposed DeepBiteNet architecture in addressing the complex task of multiclass bug bite classification. Achieving a test accuracy of 84.6%, DeepBiteNet significantly outperformed a comprehensive suite of baseline models, including both heavyweight networks such as DenseNet169 and InceptionV3, as well as lightweight mobile-friendly architectures like MobileNetV2, ShuffleNet, and SqueezeNet. This performance gain can be primarily attributed to the architectural design of DeepBiteNet, which strategically integrates heterogeneous convolutional feature extractors via a stacked meta-classifier. The backbone networks were chosen to balance representational diversity and computational efficiency. DenseNet121 offered deep feature connectivity and reuse, EfficientNet-B0 contributed compound scaling, and MobileNetV3 ensured optimization for deployment. The meta-classifier’s role in aggregating predictions proved crucial in refining classification boundaries and correcting model-specific biases. Unlike soft voting or averaging, the meta-learner demonstrated an ability to selectively amplify accurate predictions while down weighting erroneous outputs—a trait that is particularly advantageous when dealing with fine-grained visual similarities, such as between mosquito and flea bites.

The improvements were not limited to accuracy alone. DeepBiteNet also demonstrated superior performance across all additional evaluation metrics, including macro-averaged precision (0.880), recall (0.870), and F1-score (0.875). These metrics are critical for real-world applications where false positives or false negatives can misguide medical interpretation and lead to suboptimal patient management. For example, misclassifying a tick bite as benign could result in delayed treatment of Lyme disease or other tick-borne illnesses. One of the most compelling advantages of DeepBiteNet is its adaptability to mobile deployment. Unlike computationally expensive models that require high-end GPUs, the individual backbones and the ensemble meta-classifier were quantized and converted to TensorFlow Lite without significant degradation in performance. This enables on-device inference in real time, making the model accessible in rural or low-resource settings where dermatological expertise is unavailable. Nonetheless, several limitations merit consideration. First, although the dataset used in this study is publicly available and diverse, it remains modest in size. While data augmentation strategies have mitigated some of the associated generalization issues, future work would benefit from a substantially larger, clinically annotated dataset that includes multiple images per case, temporal progression, and metadata such as patient symptoms or geographic origin. Additionally, class imbalance remains a persistent challenge, particularly for less-represented categories such as tick and chigger bites. Although stratified sampling and regularization techniques were employed, long-tailed distributions can still skew learning dynamics. Another limitation lies in the model’s dependency on the visual manifestation of bites. In many real-world scenarios, insect bites may be partially healed, obscured by skin conditions, or masked by pigmentation differences, especially across varied ethnic populations. These factors can reduce model robustness and highlight the importance of incorporating multimodal inputs in future extensions, such as combining image analysis with user-reported symptoms or temporal data. While Grad-CAM and similar techniques can offer post hoc explainability, there is a need to enhance transparency by integrating inherently interpretable models or attention-guided architectures. This would improve clinical trust and facilitate broader adoption among practitioners. The DeepBiteNet model presents a promising step forward in AI-assisted insect bite classification, offering a well-balanced trade-off between accuracy, interpretability, and deployability. Its ensemble-based design, combined with strategic transfer learning and lightweight optimization, positions it as a strong candidate for integration into mobile teledermatology platforms and community health systems.

## 6. Conclusions

This study introduced DeepBiteNet, a novel ensemble-based deep learning architecture specifically designed for the multiclass classification of insect bite images. By integrating diverse convolutional neural network backbones—DenseNet121, EfficientNet-B0, and MobileNetV3—through a stacked meta-classification strategy, the proposed framework effectively captured both general and class-specific visual patterns across a heterogeneous dataset. Extensive experiments conducted on a publicly available bug bite image dataset demonstrated that DeepBiteNet outperforms 15 widely recognized baseline models in all major evaluation metrics, achieving a state-of-the-art classification accuracy of 84.6% along with high precision, recall, and F1-score values. The success of DeepBiteNet underscores the advantages of architectural heterogeneity and meta-level decision fusion in complex image classification tasks characterized by fine-grained inter-class variability. Moreover, the framework was optimized for deployment through model quantization and compression, ensuring compatibility with real-time inference on resource-constrained mobile devices—an essential feature for field applications in remote or underserved areas. While the results are promising, the study also highlights the importance of addressing data limitations. Future work will focus on expanding the dataset with clinically verified annotations, incorporating multimodal features such as symptom descriptions and location metadata, and exploring explainable AI mechanisms to increase transparency in model predictions. Additionally, efforts will be made to evaluate the model’s robustness across diverse demographic groups and skin tones, as well as to adapt the system to progressive or overlapping dermatological conditions. DeepBiteNet establishes a strong foundation for the application of ensemble deep learning in digital dermatology, particularly in the context of automated insect bite recognition. Its strong predictive performance, lightweight deployment capabilities, and scalability make it a viable solution for enhancing diagnostic support in global health contexts where dermatological expertise is limited.

## Figures and Tables

**Table 1:** Summary of representative works in insect bite and dermatological image classification.

Study	Task	Models	Dataset	Mobile-Ready	Accuracy
Ilijoski et al. (2023) [1]	Bug bite classification	VGG16 + InceptionV3	Kaggle + curated	No	~86%
Sushma & Pande (2023) [2]	Multiclass bite classification	MobileNetV2	Self-curated	No	~76%
Akshaykrishnan et al. (2023) [4]	Insect bite classification	CNN + SVM	Custom dataset	No	~78%
Asif et al. (2024) [16]	Lesion classification	SKINC-Net	Dermoscopy	Yes	83%
Amin et al. (2024) [17]	Skin disease	CNNs	Public datasets	No	86%

**Table 2:** Distribution of images across insect bite categories in the cleaned dataset.

Class	Number of Images
Ant	239
Bed Bug	218
Chigger	213
Flea	251
Mosquito	287
Spider	265
Tick	206
Unaffected Skin	253
Total	1932

**Table 3:** Classification accuracy (%) of DeepBiteNet versus baseline CNN models on the test set.

Model	Accuracy (%)	Precision	Recall	F1-Score	Params (M)	FLOPs (G)
DeepBiteNet	84.6	0.88	0.87	0.875	~15.8	~3.2
DenseNet121	78.2	0.742	0.732	0.737	7.98	2.9
DenseNet169	77.9	0.757	0.747	0.752	14.15	3.4
EfficientNet-B1	77.2	0.788	0.778	0.783	7.8	0.7
EfficientNet-B0	76.9	0.773	0.763	0.768	5.3	0.39
InceptionV3	76.3	0.665	0.655	0.66	23.9	5.7
Xception	76	0.803	0.793	0.798	22.9	8.4
ConvNeXt-T	75.9	0.865	0.855	0.86	28.6	4.5
MobileNetV3	75.4	0.727	0.717	0.722	2.5	0.07
MobileNetV2	75.1	0.711	0.701	0.706	3.4	0.3
NASNetMobile	74.6	0.819	0.809	0.814	5.3	0.6
ResNet50	74.5	0.65	0.64	0.645	25.6	4.1
VGG19	73.5	0.696	0.686	0.691	143.7	19.6
VGG16	72.8	0.681	0.671	0.676	138.3	15.5
ShuffleNet	71.5	0.834	0.824	0.829	1.4	0.14
SqueezeNet	70.8	0.849	0.839	0.844	1.2	0.24

**Table 4:** Per-class precision, recall, and F1-score for the DeepBiteNet model on the test set.

Class	Precision	Recall	F1-Score
Ant	0.88	0.87	0.875
Bed Bug	0.86	0.84	0.85
Chigger	0.83	0.82	0.825
Flea	0.89	0.90	0.895
Mosquito	0.91	0.92	0.915
Spider	0.88	0.86	0.87
Tick	0.84	0.83	0.835
Unaffected Skin	0.87	0.88	0.875

**Table 5:** Comparison of DeepBiteNet performance trained with and without data augmentation.

Setting	Accuracy (%)	Precision	Recall	F1-Score
Without Augmentation	79.2	0.81	0.80	0.805
With Augmentation	84.6	0.88	0.87	0.875

**Table 6:** Performance comparison of DeepBiteNet trained with and without ImageNet pre-trained weights.

Initialization	Accuracy (%)	Precision	Recall	F1-Score
Without Pretraining	78.5	0.79	0.78	0.785
With ImageNet Weights	84.6	0.88	0.87	0.875

## Data Availability

All datasets used are available online, with open access.
